# Application of dried blood spot sample pooling strategies for *Plasmodium* 18S rRNA biomarker testing to facilitate identification of infected persons in large-scale epidemiological studies

**DOI:** 10.1186/s12936-021-03907-8

**Published:** 2021-10-07

**Authors:** Ming Chang, Selena Johnston, Annette M. Seilie, Dianna Hergott, Sean C. Murphy

**Affiliations:** 1 Department of Laboratory Medicine and Pathology, University of Washington, Seattle, WA USA; 2 Department of Epidemiology, University of Washington, Seattle, WA USA; 3 Department of Microbiology, University of Washington, Seattle, WA USA; 4 Center for Emerging and Re-Emerging Infectious Diseases, University of Washington, Seattle, WA USA

**Keywords:** *Plasmodium*, 18S rRNA, Biomarker, qRT-PCR, Dried blood spot, Pooled testing, Graphical user interface

## Abstract

**Background:**

*Plasmodium* 18S rRNA is a sensitive biomarker for detecting *Plasmodium* infection in human blood. Dried blood spots (DBS) are a practical sample type for malaria field studies to collect, store, and transport large quantities of blood samples for diagnostic testing. Pooled testing is a common way to reduce reagent costs and labour. This study examined performance of the *Plasmodium* 18S rRNA biomarker assay for DBS, improved assay sensitivity for pooled samples, and created graphical user interface (GUI) programmes for facilitating optimal pooling.

**Methods:**

DBS samples of varied parasite densities from clinical specimens, *Plasmodium falciparum *in vitro culture, and *P. falciparum* Armored RNA® were tested using the *Plasmodium* 18S rRNA quantitative triplex reverse transcription polymerase chain reaction (qRT-PCR) assay and a simplified duplex assay. DBS sample precision, linearity, limit of detection (LoD) and stability at varied storage temperatures were evaluated. Novel GUIs were created to model two-stage hierarchy, square matrix, and three-stage hierarchy pooling strategies with samples of varying positivity rates and estimated test counts. Seventy-eight DBS samples from persons residing in endemic regions with sub-patent infections were tested in pools and deconvoluted to identify positive cases.

**Results:**

Assay performance showed linearity for DBS from 4 × 10^7^ to 5 × 10^2^ parasites/mL with strong correlation to liquid blood samples (r^2^ > 0.96). There was a minor quantitative reduction in DBS rRNA copies/mL compared to liquid blood samples. Analytical sensitivity for DBS was estimated 5.3 log copies 18S rRNA/mL blood (28 estimated parasites/mL). Properly preserved DBS demonstrated minimal degradation of 18S rRNA when stored at ambient temperatures for one month. A simplified duplex qRT-PCR assay omitting the human mRNA target showed improved analytical sensitivity, 1 parasite/mL blood, and was optimized for pooling. Optimal pooling sizes varied depending on prevalence. A pilot DBS study of the two-stage hierarchy pooling scheme corroborated results previously determined by testing individual DBS.

**Conclusions:**

The *Plasmodium* 18S rRNA biomarker assay can be applied to DBS collected in field studies. The simplified *Plasmodium* qRT-PCR assay and GUIs have been established to provide efficient means to test large quantities of DBS samples.

**Supplementary Information:**

The online version contains supplementary material available at 10.1186/s12936-021-03907-8.

## Background

Despite increased investment and efforts in malaria control, malaria continues to be a major cause of morbidity and mortality in endemic countries [1]. *Plasmodium* infections are commonly diagnosed by microscopy or rapid diagnostic tests (RDTs). The limit of detection (LoD) for blood smear microscopy varies from 5 to 50 parasites/µL blood depending on experience and training of the technologists [2–5]. RDTs that detect histidine rich protein 2 (HRP2) from *Plasmodium falciparum* and/or lactate dehydrogenase (LDH) expressed by all human-infecting *Plasmodium* species have estimated LoDs of 200–2000 parasites/µL [6], although a more sensitive RDT is also available with a LoD of ~ 1 parasite/µL [7]. Recent analyses using much more sensitive molecular assays, some of which detect as few as 10–20 parasites/mL (0.01–0.02 parasites/µL) of blood [8, 9], showed that RDTs and microscopy detected less than half of all *P. falciparum* infections [10]. Epidemiology studies have determined that *Plasmodium* transmission from an infected person to a mosquito can occur even at these low densities [11–13]. The use of molecular assays has greatly informed the understanding of malaria epidemiology, especially for asymptomatic, sub-microscopic infections [14–16], which may improve effectiveness of interventions like vector control, mass drug administration, and vaccination, but the distribution and dynamics of such infections in endemic countries is largely unknown, and additional studies using highly sensitive molecular assays are needed. However, molecular assays (i.e., polymerase chain reaction (PCR) and reverse transcription PCR (RT-PCR) for the *Plasmodium* 18S rDNA or rRNA, respectively) are considered high complexity tests, and expanding their use to inform larger field studies can be constrained by limited availability of molecular assay platforms, the need for venous blood collection procedures, sample storage and stability, and testing costs.

Dried blood spots (DBS) are a practical sample type for large population studies and do not require venous blood collection, making them well suited for resource-limited regions. Nucleic acids spotted onto DBS are stable at ambient temperature when desiccated [17], making DBS favorable for collection, transportation, and storage. Numerous PCR [18–22] and RT-PCR [23, 24] assays have thus relied on DBS for malaria studies. The *Plasmodium* 18S rRNA qRT-PCR developed by the University of Washington (UW) is particularly well-suited to DBS since the abundance of the 18S rRNA itself means that even a single ring-stage *P. falciparum* parasite can be detected from one 50 µL DBS [8]. Moreover, a laser cutting device was established and utilized to excise spots from DBS cards to eliminate the potential for contact-based cross contamination [25].

Malaria epidemiological studies often generate a very large number of samples [2, 26, 27], and as the prevalence of infection decreases, more samples need to be tested to identify infected persons. As such, laboratory testing for pooled samples is appealing since pooling could potentially lower reagent costs, reduce labour, and accelerate results by reducing laboratory test counts [28, 29]. Optimal pooling strategies depend on the prevalence, expected range of parasite densities [30], and analytical sensitivity of the molecular assays in use.

To address issues described earlier, this study evaluated key steps of laboratory testing to carry out epidemiological studies. *Plasmodium* 18S rRNA qRT-PCR used in this study (UW qRT-PCR), a laboratory-developed test run on the Abbott m2000 platform to detect the *Plasmodium* 18S rRNA /rDNA biomarker in controlled human malaria infection (CHMI) studies in non-endemic sites, was rigorously reviewed by the FDA [31]. Here, this study evaluted performance of the UW qRT-PCR assay for a different use case—DBS, investigated various pooling strategies to reduce labour and reagent costs in varied malaria prevalence settings, developed an optimal method for pooled DBS samples, and validated one such pooling strategy against individually tested samples.

## Methods

### Standards and controls

Quantitative standards used Armored RNA® containing *P. falciparum* 18S rRNA (*P. falciparum* Armored RNA®, Asuragen) at 7.7, 6.7, 5.7 and 4.7 log_10_ copies per mL in whole blood lysate. Whole blood lysates were made at a ratio of 25 µL EDTA whole blood to 1 mL NucliSENS® lysis buffer (bioMérieux, Marcy-l'Etoile, France). The standards were equivalent to 2.9 × 10^5^, 2.9 × 10^4^, 2.9 × 10^3^, and 2.9 × 10^2^ estimated *P. falciparum* ring-stage parasites/mL blood, respectively, based on a previously determined conversion factor [8, 25].

To evaluate assay performance, *P. falciparum* 3D7 parasites cultured in vitro, synchronized as ring stages, and counted for parasitaemia were used to prepare controls. They were diluted in *Plasmodium*-negative EDTA whole blood to nominal densities of 4 × 10^7^, 5 × 10^5^, 5 × 10^4^, 5 × 10^3^, and 5 × 10^2^ parasites/mL for both liquid samples and DBS. Both liquid and DBS samples were tested to compare linearity and estimate quantitative bias between sample types. Additional DBS samples were prepared at nominal densities of 7 × 10^4^ and 7 × 10^2^ parasites/mL, and 20 replicates of each as well as *Plasmodium*-negative DBS were tested over ten days to evaluate within- and between-run precision following the same way as the previous publication [8].

To evaluate sensitivity of the standard triplex and simplified duplex assays in terms of 18S rRNA copy number, *P. falciparum* Armored RNA® were diluted in EDTA blood to nominal 9.2, 8.3, 7.3, 6.3, 5.3, 5.1 and 4.7 log_10_ copies/mL blood, and spotted to DBS or added to lysis buffer. In addition, leftover, de-identified EDTA whole blood samples from participants in *P. falciparum* CHMI trials were spotted to DBS cards and added to lysis buffer to evaluate differences in sample types.

### Preparation of DBS and liquid samples

Liquid samples described in this study were 50 µL EDTA whole blood added to 2 mL NucliSENS® lysis buffer. DBS samples were 50 µL of EDTA whole blood spotted onto Whatman Protein Saver 903 Snap-apart card papers (GE/Whatman, Kent, UK). DBS samples were air-dried in biosafety hoods for at least four hours and preserved individually in gas-impermeable plastic bags containing a 3-Spot—30%, 40%, and 50%—humidity indicator card (Desco Industries Inc, Chino, CA) and desiccant packets (Fisher Scientific, Pittsburgh, PA). DBS and liquid samples were moved to -80 °C within 24 h of preparation. At the time of testing, DBS were excised from cards using a laser cutter [25] into a receiver tube of at least 13 mm in diameter. Excised spots were immersed in 2 mL lysis buffer (named DBS lysate herein) and incubated at 55 °C for 30 min, with brief shaking every 10 min. Dilution of DBS samples or standards was carried out by mixing DBS lysates with negative DBS lysate depending on dilution factors. Tubes were centrifuged for 10 min at 2000 × *g* at 25 °C to deposit spots at the bottom of tubes and reduce bubbles and foaming [32]. At the time of testing, liquid samples were thawed at room temperature, vortexed, and centrifuged at 2000 × *g* for 5 min at 25 °C.

To examine biomarker stability in DBS, DBS samples containing dilutions of *P. falciparum* culture at 3.5 × 10^6^, 1.2 × 10^4^, 1.4 × 10^3^ or 5 × 10^2^ parasites/mL were preserved in standard conditions described earlier and placed in a − 80 °C freezer, a − 20 °C freezer, a laboratory bench (i.e., 22 ± 2 °C with 55% humidity) or an incubator (i.e., 37 °C with 30% humidity) for one month. The 30% spot on the humidity indicator card packed with DBS remained blue (i.e., desiccated) for all samples. Three DBS spots for each condition were subsequently tested in duplicate on different days by the standard triplex *Plasmodium* 18S qRT-PCR assay.

### *Plasmodium* 18S rRNA real-time quantitative RT-PCR assay

A detailed procedure of this assay was previously described [8]. In brief, one mL lysate of DBS lysates and liquid samples was extracted for RNA using the Abbott m2000sp instrument (Abbott Molecular, Niles, IL). Quantitative RT-PCR (qRT-PCR) was performed on an Abbott m2000rt. The triplex qRT-PCR assay used a Bioline SensiFAST™ Probe LO-ROX One-Step Kit (Bioline, London, UK) and three sets of primers and probes to amplify *P. falciparum* 18S rRNA (*P. falciparum* qRT-PCR), pan-*Plasmodium* 18S rRNA (Pan qRT-PCR), and a mRNA encoding human TATA binding protein (TBP RT-PCR). Cycling conditions were 48 °C for 10 min and 95 °C for 2 min, followed by 45 cycles at 95 °C for 5 s and 50 °C for 35 min. Individual DBS samples were tested using the triplex qRT-PCR, called the ‘standard’ triplex assay. During pooled testing, human TBP-specific reagents were omitted, and this was named the ‘simplified’ duplex assay. Primer and probe concentrations used for qRT-PCR and sequences are described in Additional file 2: Text S2.

### Estimating total test counts for two-stage hierarchical, square matrix, and three-stage hierarchical pooling schemes

To evaluate three pooling schemes commonly mentioned in epidemiological studies on infectious diseases regarding the numbers of laboratory tests, GUIs were designed to generate best estimation of total test counts for each scheme. In brief, pooling organizes multiple samples together into pools of varied sizes, and tests for the presence of the biomarker in the pool. If a pool is negative for the biomarker, this indicates that all samples in the pool are negative. If a pool is positive, all samples in the positive pool were subsequently tested individually (deconvolution). GUI calculated total test counts for two parts of the testing procedure: the number of tests required to screen pools and the number of tests for deconvolution.

The commonly called *two-stage hierarchical pooling scheme* originated from Dorfman’s minipool algorithm [33]. For epidemiological studies of estimated prevalence rate (*x*), the optimal pooling size was approximated to be *x*^*−*0.5^ [29]. A GUI (Additional file 3: File S1) was designed to estimate the total test number for *y* samples collected from studies of an estimated prevalence rate. In addition, a graph displayed total test counts for pooling size of *x*^−0.5^ ± 1, *x*^−0.5^ ± 2, and *x*^−0.5^ ± 3. As the number of samples and the prevalence rate are entered, the optimal pooling size is calculated, and test counts for pools and deconvolution are subsequently summed to yield the lowest total test count required to identify positive samples in the cohort. Estimates represent maximum test counts because the program assumes that positive samples are evenly distributed among pools.

A *square matrix pooling scheme* consisted of an identical number of rows and columns and was first described for rapid identification of positive cases in the array [34–36]. The pool size was N^2^ for a matrix of N rows and N columns. A GUI (Additional file 3: File S2) was designed to allow users to manipulate the matrix of N to yield total test counts after entering the number of samples and the predicted prevalence. The accompanying graph shows additional total test counts for matrices of N ± 1, N ± 2, and N ± 3 and guides users to search for the optimal pooling size yielding the smallest total test count. Calculating total test counts is described in Additional file 1: Text S1. The total test count is the sum of the screening tests (2 N) plus the number of tests to deconvolute all positive pools.

The *three-stage hierarchical pooling strategy* [29] consisted of a two-step pooling process. A GUI designed here (Additional file 3: File S3) allowed users to manipulate sizes of the first and the second pooling steps after the number of samples and predicted prevalence were entered. The accompanied first graph guides users to choose an optimal pooling size for the second step pooling and the second graph displays the total test counts given for the first and second pooling sizes. The total test counts calculated by Additional file 3: File S3 are potential maximum test counts for given cohorts assuming that all positive samples are evenly distributed in the first pools. The presented trend line in the bottom graph indicating maximum total test counts helps users choose different pooling sizes to identify the lowest total test number for each prevalence rate.

### Pilot demonstration of a two-stage hierarchy pooling strategy using archival DBS samples

DBS collected from asymptomatic participants in an IRB-approved field study conducted in Zanzibar, Tanzania were shipped to the UW Malaria Molecular Diagnostic Laboratory at ambient temperature and stored at -80 °C. One hundred and seven individual DBS from this cohort were previously tested with an earlier version of the *Plasmodium* 18S rRNA RT-PCR assay [8] and showed an 8% positivity rate. The initial testing was performed within 14 months of sample collection. At this 8% positivity rate, total test counts were determined to be similar among the three aforementioned pooling methods. The two-stage hierarchy pooling strategy was chosen because the upfront sample pooling maneuver required the fewest laboratory-based steps. Seventy-eight DBS archived over a three-year period were retrieved from − 80 °C storage and were blindly tested using mini pools of n = 3 distinct samples/pool. Individual DBS lysate was prepared as described earlier and then 0.5 mL of each three DBS lysates were transferred to a ‘pool’ tube; each pool tube consisted of three distinct samples. Pool tubes were extracted and amplified as described above. The remainder of each DBS lysate was stored at 4 °C to facilitate deconvolution as needed. All 26 pools were tested on the first day, and positive pools were deconvoluted by testing each residual DBS lysates of pooled constituent samples the following day.

### Statistics

GraphPad Prism version 8.4.1 was used to calculate a best-fit non-linear regression equation, plot the Bland–Altman biased test and graphs. Welch’s *t* test was used to analyse differences between groups.

## Results

### Biomarker testing on DBS samples

**Fig. 1:**
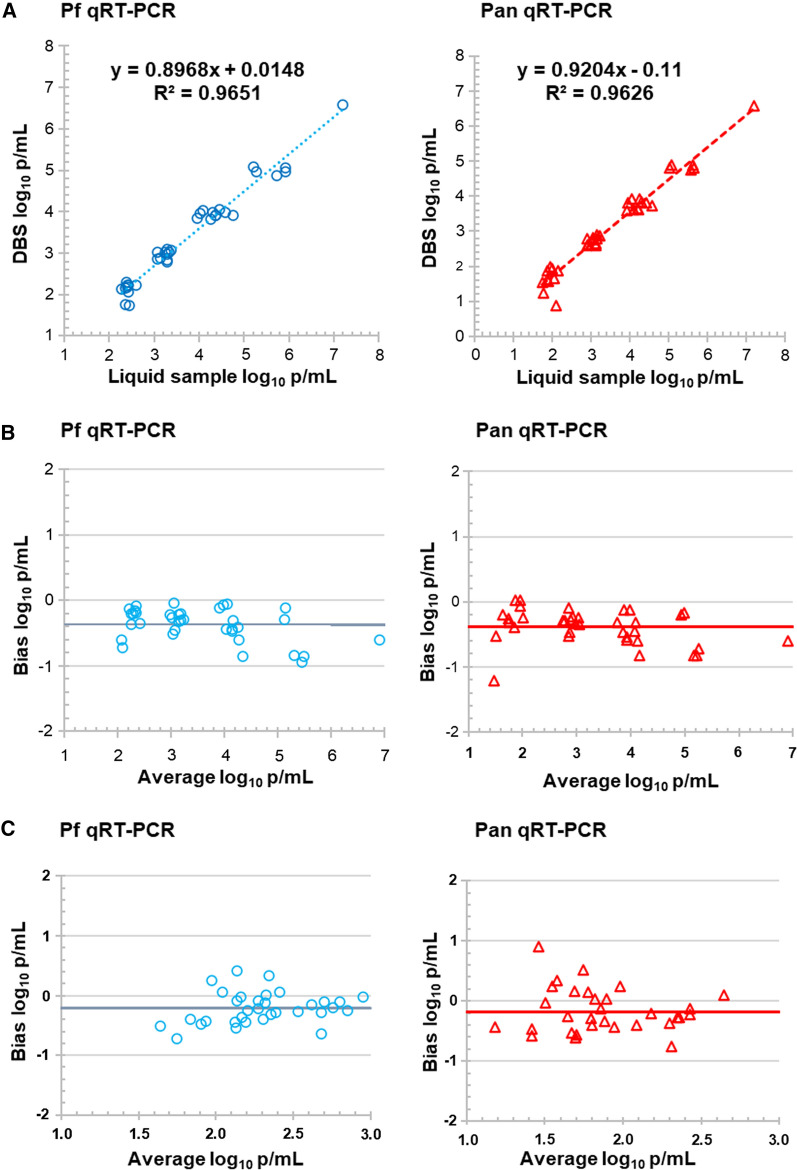
*Plasmodium* 18S rRNA biomarker correlation between DBS samples and liquid samples. Serial dilutions of *P. falciparum* cultures containing 4 × 10^7^, 5 × 10^5^, 5 × 10^4^, 5 × 10^3^ and 5 × 10^2^ parasites/mL and 1, 5, 10, 10, and 10 replicates were used to prepare DBS and liquid samples and tested by the standard assay (**A**, **B**). The correlation between liquid and DBS samples were > 0.96 (**A**). Bland–Altman plots displayed biases (DBS-liquid samples) < 0.4 log_10_ parasites/mL blood (**B**). Leftover clinical samples were collected from seven infected individuals throughout the course of their treatment to prepare DBS and liquid samples (**C**). Bland–Altman plots displayed biases close to 0.2 log_10_ parasites/mL blood. *Pf* *P. falciparum*, *Pan* pan-*Plasmodium*

Linearity of the qRT-PCR for DBS samples in the range from 5 × 10^2^ to 4 × 10^7^ parasites/mL was compared to liquid samples. Correlation between liquid and DBS samples was strong (r^2^ > 0.96) (Fig. 1A), indicating linearity of the assay for DBS samples. Bland–Altman (difference) plots indicated that DBS results yielded quantitative results slightly lower than liquid samples (Fig. 1B) with bias of − 0.37 and − 0.39 log_10_ estimated parasites/mL blood by the *P. falciparum* qRT-PCR and Pan qRT-PCR, respectively (Table 1). For infected patient samples, Bland–Altman plots showed a similar trend for DBS *versus* liquid samples with bias of − 0.21 and − 0.18 log_10_ estimated parasites/mL blood by the *P. falciparum* qRT-PCR and Pan qRT-PCR, respectively (Fig. 1C and Table 1). Overall, the 95% confidence interval (CI) for such differences was < 1.0 log_10_ parasites/mL blood.

**Table 1:** Quantitative bias for DBS samples compared to liquid samples

Sample type	*Pf* qRT-PCR		Pan qRT-PCR	
	Bias (log_10_ parasites/mL blood)	95% limits of agreement	Bias (log_10_ parasites/mL blood)	95% limits of agreement
Serial dilutions of *Pf* cultures	− 0.37	− 0.86–0.12	− 0.39	− 0.91–0.14
Venous blood of infected individuals	− 0.21	− 0.73–0.31	− 0.19	− 0.93–0.55

*Pf P. falciparum*, *Pan* pan-*Plasmodium*

The means for 20 DBS replicas of the high positive control measured by *P. falciparum* qRT-PCR and Pan qRT-PCR were 8.7 log_10_ copies/mL blood and 8.6 log_10_ copies/mL blood, respectively (Table 2), and within-lab CVs were 3.32 and 3.99%, respectively. The means of 20 DBS replicas for the low positive control quantified by *P. falciparum* qRT-PCR and Pan qRT-PCR were 6.7 log_10_ copies/mL blood and 6.3 log_10_ copies/mL blood, respectively, and within-lab CVs for the low positive control were 4.07% and 5.61%, respectively. All negative DBS were undetectable by qRT-PCR.

**Table 2:** Assay precision for DBS samples. Each result generated as copies/mL DBS lysate, converted log copies/mL blood to calculate within-run and within-lab percent coefficient of variation (% CV) and 95% confidence interval (95% CI)

qRT-PCR	Control	N (days)	Mean log_10_ copies/mL blood	Within-run % CV (95% CI)	Within-lab %CV (95% CI)
*Pf*	High positive	20 (10)	8.66	0.97% (0.68–1.71%)	3.32% (2.52–4.85%)
	Low positive	20 (10)	6.69	2.73% (1.91–4.79%)	4.07% (3.10–5.95%)
Pan	High positive	20 (10)	8.62	1.10% (0.77–1.93%)	3.99% (3.03–5.83%)
	Low positive	20 (10)	6.32	3.29% (2.30–5.77%)	5.61% (4.26–8.19%)

*Pf P. falciparum*, *Pan* pan-*Plasmodium*

### Stability of biomarker in DBS samples

Several desiccants included in each sealed package were sufficient to maintain humidity < 30% to preserve one DBS card for one month either stored at − 80 °C, − 20 °C, 22 °C or 37 °C. Quantities of *P. falciparum* 18S rRNA in DBS analysed by Welch’s *t*-test showed statistically significant differences in quantitative results for DBS stored at 37 °C *versus* − 80 °C (Table 3). Means (± SEM) of such differences were -0.4 (± 0.2) and − 0.2 (± 0.1) log_10_ copies/mL blood for the high and moderate controls, respectively, measured by pan-*Plasmodium* qRT-PCR (Additional file 4: Fig. S1). The difference in DBS stored at 22 °C vs. − 80 °C was also statistically significant for the moderate level DBS but not for the high-level DBS. Differences were not significantly different for the low and very low levels DBS (p > 0.2). Analyses by Welch’s *t* test demonstrated that 18S rRNA levels in all DBS stored at − 20 °C were no different than those stored at − 80 °C (p > 0.1).

**Table 3:** Stability of *P. falciparum* culture 18S rRNA in DBS stored at four different temperatures

DBS control level	Estimated parasites/mL	Log_10_ copies/mL blood	*Pf* qRT-PCR			Pan qRT-PCR		
			*P* value by Welch’s *t*-test for log_10_ copies/mL blood					
			− 20 °C vs.− 80 °C	22 °C vs. − 80 °C	37 °C vs. − 80 °C	− 20 °C vs. − 80 °C	22 °C vs. − 80 °C	37 °C vs. − 80 °C
High	3.5 × 10^6^	10.41	0.719	0.340	**0.034**	0.647	0.441	**0.037**
Moderate	1.2 × 10^4^	7.95	0.376	**0.012**	0.077	0.362	**0.013**	**0.044**
Low	1.4 × 10^3^	7.02	0.207	0.637	0.935	0.292	0.874	0.330
Very Low	5 × 10^2^	6.57	0.812	0.716	0.457	0.694	0.804	0.558

*Pf P. falciparum*, *Pan* pan-*Plasmodium*

Bold values denote *p* values < 0.05

### Quantifying serial dilutions of *P. falciparum* Armored RNA® in DBS

During the pooling procedure, *Plasmodium* 18S rRNA molecules are released from the DBS matrix and pooled for testing. To mimic the dilution of parasite-derived 18S rRNA in pooled samples, the *P. falciparum* Armored RNA® was diluted in whole blood, and then spotted onto DBS or added to lysis buffer to generate paired liquid samples. Quantitative measurements and overall detection were compared across seven concentration levels of *P. falciparum* Armored RNA® in liquid and DBS (Additional file 4: Table S1). Liquid samples were deemed to represent 100% recovery, and DBS results were compared to such paired liquid sample results. The standard biomarker assay was positive for all liquid samples at concentrations down to 5.1 log_10_ copies/mL, consistent with previous observation [8], and down to 5.3 log_10_ copies/mL blood for DBS samples (Additional file 4: Table S1).

The standard triplex qRT-PCR includes a human TBP mRNA target to monitor integrity of each sample. However, in pooled samples, TBP results do not reflect the condition of each individual DBS sample in the pool. Since the analytical sensitivity of the DBS assay was reduced compared to the sensitivity of liquid samples and since the TBP mRNA control was less informative for pooled testing, a simplified duplex qRT-PCR omitting the TBP reagents was also evaluated for serial dilutions of *P. falciparum* Armored RNA® (Table 4). Analytical sensitivity was improved and all liquid samples and 90% of DBS were detected at a nominal density of 4.7 log_10_ copies/mL (~ 7 estimated parasites/mL).

**Table 4:** Measurements and percentages of detection rates for varied concentrations of *P. falciparum* Armored RNA® in liquid and DBS samples by the simplified assay

*Pf* Armored RNA® Dilution		Liquid samples (log_10_ copies/mL blood)				DBS samples (log_10_ copies/mL blood)			
Parasites/mL blood	Log_10_ copies/mL blood	*Pf* qRT-PCR		Pan qRT-PCR		*Pf* qRT-PCR		Pan qRT-PCR	
		Mean	SD	Mean	SD	Mean	SD	Mean	SD
2.4 × 10^5^	9.2	9.0	0.2	8.9	0.3	8.6	0.3	8.6	0.3
2.8 × 10^4^	8.3	8.5	0.2	8.0	0.2	7.7	0.4	7.7	0.3
2,8 × 10^3^	7.3	6.9	0.1	7.0	0.2	6.7	0.3	6.6	0.2
2.8 × 10^2^	6.3	5.9	0.1	5.9	0.2	5.6	0.4	5.7	0.3
2.8 × 10^1^	5.3	4.8 (3/4*)	n/a	4.9	0.2	4.8 (6/8*)	n/a	4.8	0.3
1.7 × 10^1^	5.1	4.8 (3/4*)	n/a	4.8	0.2	4.7 (6/10*)	n/a	4.6	0.4
7	4.7	0.0 (0/4*)	n/a	4.1	0.3	3.4 (5/11*)	n/a	3.2 (9/11*)	n/a

*Pf P. falciparum*, *Pan* pan-*Plasmodium*

*n/a* not applicable (SD not calculated for replicates of < 100% detection rate)

SD reflects variation in two batches of samples measured by multiple operators; n = 4–11 samples per concentration level

*Average calculated for quantifiable samples only (numbers in parentheses are detected results/number of replicates tested)

### Diluting DBS samples to mimic the pooling process

To address performance of the standard assay and the simplified assay for low density samples following pooling with negative samples, DBS of *P. falciparum* Armored RNA® Dilutions containing equivalent 7.3, 6.3, 5.3and 5.1 log_10_ copies/mL blood, respectively, were tested after 3- and tenfold dilutions (Additional file 4: Table S2). Undiluted samples, 1:3 dilutions, and 1:10 dilutions were tested by the standard and simplified assays. Measurements were similar for Level C and Level D (nominal 7.32 and 6.32 log_10_ copies/mL blood, respectively) of *P. falciparum* Armored RNA® DBS by the standard triplex and simplified duplex assays. However, the simplified assay achieved better sensitivity for *P. falciparum* Armored RNA® DBS containing lower copy numbers (the third column of Additional file 4: Table S2).

To mimic a low-density sample pooled amongst many negative samples, a DBS sample prepared from EDTA blood containing 250 parasites/mL from an infected human individual was serially diluted and tested by the duplex assay. After the spot was excised and incubated with 2 mL lysis buffer, the lysate was further diluted in serial fourfold dilutions. Testing was carried out by the simplified duplex assay (Table 5) and detected as few as 800 copies of 18S rRNA per 50 µL spot and exhibited a linear range from undiluted through the 1:256 dilution.

**Table 5:** Dilutions of clinical DBS containing *P. falciparum* measured by the simplified duplex assay

Dilution	Nominal concentration			*Pf* qRT-PCR		Pan qRT-PCR	
	Parasites/mL blood	Log_10_ copies/mL blood	Log_10_ copies/spot	Parasites/mL blood	Log_10_ copies/spot	Parasites/mL blood	Log_10_ copies/spot
Neat	2.5 × 10^2^	6.3	5.0	1.9 × 10^2^	4.9	5.9 × 10^2^	5.3
1:4	6.3 × 10^1^	5.7	4.4	4.0 × 10^1^	4.2	9.6 × 10^1^	4.6
1:16	1.6 × 10^1^	5.1	3.8	1.0 × 10^1^	3.6	2.5 × 10^1^	4.0
1:64	4	4.5	3.2	1.8 × 10^1^	3.8	8	3.5
1:256	1	3.9	2.6	ND	ND	2	2.9

*Pf P. falciparum*. *Pan* pan-*Plasmodium*

*ND* not detected

### Modelling of three potential malaria DBS pooling strategies

Based on the need to identify adequate batch testing for large numbers of samples, GUI programs were designed for three common pooling schemes – two-stage hierarchy, square matrix pooling, and three-stage hierarchy. These GUIs allowed the laboratory staff to search and identify optimal pooling strategies for provided samples by entering the number of samples and the estimated prevalence of the studied cohort. The following demonstrate outputs of each GUI program.

For the two-stage hierarchy pooling scheme, the optimal pooling size (x^−1/2^) was dependent on the prevalence rate (*x*) (Additional file 3: File  S1). Theoretical cohorts of 1000 samples with prevalence rates of 0.1, 0.5, 2, 5 and 10% were evaluated by the two-stage hierarchy pooling strategy. For 0.1, 0.5, 2, 5, and 10% prevalence rates, a pool size of 28, 14, 7, 4–5, and 3 samples/pool yielded the lowest count of 36, 142, 283, 450, and 643 tests, respectively. Additional file 4: Fig. S2A shows a visual comparison for varied pooling sizes yielding different total test counts.

The square matrix pooling scheme is an alternative pooling approach with the potential advantage of bypassing deconvolution if the average number of positive cases is ≤ 1/pool. At a low positivity rate, a positive sample can theoretically be determined by its position in the matrix (Additional file 4: Fig. S3A). If the average positivity is > 1 positive event/pool, scenarios with two or three positive cases per matrix could be evenly distributed or clustered in specific rows or columns. In the case of even distribution, a larger number of tests are required to deconvolute compared to situations in which clustered positives are present (Additional file 4: Fig. S3B vs. S3C). If the average positive is > 3/pool, the number of tests for deconvolution is significantly higher, thus smaller pooling sizes may be more efficient testing choices. This GUI (Additional file 3: File S2) allows users to identify the smallest number of total test counts by choosing the optimal pooling size while calculating the maximum total test counts. For 1000 theoretical samples at prevalence of 0.1, 0.5, 2, 5, and 10%, a pool size of 361 (19 × 19), 225 (15 × 15), 49 (7 × 7), 16 (4 × 4) and 9 (3 × 3) yielded the lowest count of 114, 157, 286, 500, and 667 tests, respectively (Additional file 4:Fig. S2B). For the square matrix pooling scheme, a pool of nine samples, for example, caused specimens to be diluted threefold whereas for the two-stage hierarchy pooling scheme, samples would be diluted ninefold. Specimen dilution factors in pools are impacted by assay sensitivity and pathogen density in the studied cohort.

**Fig. 2:**
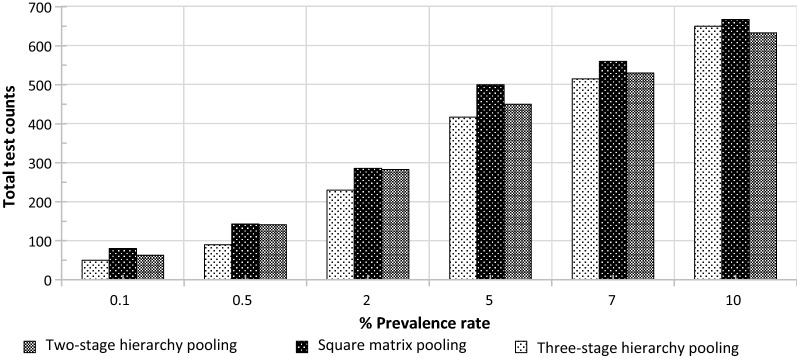
Comparisons of maximum total test counts among two-stage hierarchy, square matrix, and three-stage hierarchy pooling schemes. The smallest total test counts were estimated for each pooling strategy and graphed for a cohort of 1000 samples with predicted prevalence rates at 0.1, 0.5, 2, 5, 7% and 10%

The three-stage hierarchy pooling schemes has advantages for cohorts containing a large number of samples with low positivity rates. Because in these situations, many first-step pools and some second-step pools are expected to yield negative results, and the number of tests for deconvolution is lower compared to those required by two-stage hierarchy pooling methods. For example, this GUI (Additional file 3: File S3) determined the prevalence ranging from 0.1% to 10% for 1000 samples resulted in varied first step pool sizes from 25/pool to 4/pool (Additional file 4: Table S3). Variation of pooling sizes for 1000 samples ranging from 0.1 to 10% prevalence in the second step was narrower, between 2–5 samples/pool. The estimated lowest total test counts for 1000 samples of prevalence at 0.1, 0.5, 2, 5, 7 and 10%, were 50, 90, 230, 417, 515 and 650, respectively. When these three pooling strategies were compared (Fig. 2), there was < 10% difference in total test counts for prevalence of 7–10%. However, as prevalence dropped (i.e., 0.1, 0.5, 2, and 5%), the three-stage hierarchy pooling led to lowest total test counts.

The aforementioned calculation of maximum total test counts assumed that positive samples are evenly distributed among all samples. However, in epidemiological studies, positive samples are likely to be clustered when the samples are collected. For the three-stage hierarchy pooling schemes, a model calculated the total test counts required under the assumption that positive samples are clustered in half of the first pools and that half of the first pools also test negative. These results led to fewer number of potential samples required for second step pooling and a smaller quantity of second pools for testing. Therefore, test counts were reduced ranging from 15 to 49% for prevalence from 0.1 to 10% (Additional file 4: Table S4). Similarly, for the two-stage hierarchy pooling schemes, assuming positive samples were distributed in half of the pools, only half of potential positive pools were consequently required for deconvolution. The overall reduction of test counts ranged from 22 to 26% for prevalence from 0.1 to 10% (Additional file 4: Table S4).

### Application of a two-stage hierarchy pooling scheme for DBS samples collected from a malaria-endemic field site

Seventy-eight DBS with known results from previous individual DBS testing yielded a positivity rate of ~ 8%. The two-stage hierarchy scheme with a pool size of 3/pool was chosen after consideration of simple up-front preparation for pooled samples and a < 10% difference in total test counts among three different pooling strategies. Five of 26 pools tested positive, and 21 pools tested negative by the simplified assay (Table 6). Deconvolution of five positive pools yielded three *P. falciparum*-positive samples and two positive non-*P. falciparum* results, all of which were consistent with previous individual testing results. In addition, this pooling scheme used 41 total tests to identify five positive cases among 78 samples, reducing reagent use by approximately half compared to individual testing.

**Table 6:** Identification of positive DBS samples by a two-stage hierarchy pooling scheme (pool size = 3)

Pool ID	Result of each pool (log_10_ copies/mL blood)		Sample ID	Result of each member (log_10_ copies/mL blood)		Previously reported result
	*Pf* qRT-PCR	Pan qRT-PCR		*Pf* qRT-PCR	Pan qRT-PCR	
1–15, 17, 19, 20, 22, 23, 26	ND	ND	n/a	n/a	n/a	ND
Pool 16	5.35	5.43	Z076	ND	ND	ND
			Z077	ND	ND	ND
			Z078	5.97	6.16	*Pf*
Pool 18	3.87	3.57	Z082	ND	ND	ND
			Z083	ND	ND	ND
			Z084	3.87	4.17	*Pf*
Pool 21	ND	5.65	Z093	ND	ND	ND
			Z094	ND	6.27	Non-*Pf* *Plasmodium* sp.
			Z095	ND	ND	ND
Pool 24	ND	5.43	Z102	ND	6.03	Non-*Pf* *Plasmodium* sp.
			Z103	ND	ND	ND
			Z104	ND	ND	ND
Pool 25	5.02	5.23	Z105	ND	ND	ND
			Z106	ND	ND	ND
			Z107	5.41	5.86	*Pf*

*Pf P. falciparum*, *Pan* pan-*Plasmodium*

*ND* not detected, *n/a* not applicable

## Discussion

This study considered the application of a malaria biomarker for pooled DBS collected from epidemiological studies of natural infection or malaria prevention. Given differences in sample collection between DBS and liquid blood, DBS was regarded as a different specimen matrix requiring unique validation to test for *Plasmodium* 18S rRNA. The UW qRT-PCR assay showed strong linearity between DBS and liquid samples from 2 to 7 log_10_ parasites/mL blood. There was a small reduction in the DBS-based quantifications independent of nominal parasite densities. The reduced measurement was likely due to a small portion of nucleic acid remaining bound to DBS filter paper, as previously described [7, 37, 38]. Consequently, DBS sample quantification was also slightly less precise than seen in previous evaluations of liquid samples [8]. However, an observed within-lab standard deviation for this malaria biomarker assay was < 0.35 log_10_ copies 18S rRNA/mL blood, which is comparable to the precision reported for other DBS molecular assays that have undergone regulatory review like the CE-marked Abbott RealTime HIV-1 assay for DBS samples [32]. The streamlined procedure of the biomarker assay including laser cutting DBS, incubation of DBS in lysis buffer at 55 °C for 30 min, automated nucleic acid extraction by the Abbott *m*2000 platform and qRT-PCR amplification has shown acceptable analytical sensitivity (≥ 28 parasites/mL blood).

The described procedure utilizes two automatic instruments: one to first excise multiple spots from DBS cards and a second to then extract nucleic acids from many sample tubes simultaneously. Other investigators have shown that these steps may also be carried out manually without use of automated instruments [19].

Stability of *Plasmodium* 18S rRNA in DBS was evaluated here to consider the conditions encountered by field samples transported to central laboratories for storage and later testing. This study demonstrated that a small degree of degradation was observed in DBS 18S rRNA after one-month storage at 22 and 37 °C. Compared to DBS stored at − 80 °C freezer, DBS 18S rRNA stored in a − 20 °C freezer were equally stable for one month. Thus, DBS preserved at < 30% humidity can be transported at ambient temperatures within one month of collection with minimal degradation of 18S rRNA.

Pooled testing is a common means of handling large quantities of samples, however, issues related to assay sensitivity for diluted samples and pool sizes are often unique to the system [19, 28, 39]. This study first investigated options to improve sensitivity of a *Plasmodium* qRT-PCR assay specifically applied to pooled DBS samples. When screening pooled samples, monitoring pooled blood TBP mRNA levels does not reflect the condition of individual constituent DBS samples. qRT-PCR without targeting human TBP mRNA improved detection for *P. falciparum* Armored RNA® to 4.7 log_10_ copies/mL blood (Table 4), equivalent to ~ 0.34 parasite per 50-µL blood spot. This version of the assay was also able to detect dilutions of clinically collected DBS from 250 parasites/mL to 1 parasite/mL blood (Table 5). The sensitivity reported here is significantly improved compared to previously published methods for pooling malaria field samples [40]. The simplified duplex assay is well-suited to testing pooled DBS samples and maintains excellent sensitivity in such pools.

Studies examining the pooling strategies for human pathogens other than *Plasmodium* spp. have often used stringent and standardized FDA-approved nucleic acid amplification tests (NAAT) with fixed LoDs [29, 41]. One critical concern is the loss of NAAT sensitivity for diluted positive specimens in pools. Westreich et al*.* [29] first completed theoretical calculations for pooling algorithm sensitivities, optimal pool sizes and positive predicted values for two-stage hierarchy, three-stage hierarchy and square matrix pooling methods. Two reports [36, 42] went into greater depth by establishing a web-based application to identify optimal testing configurations based on parameters of prevalence of studied cohorts, sensitivity, and specificity of the NAAT used. Since no FDA-approved molecular assays are available to test *Plasmodium* spp., malaria molecular testing relies on laboratory-developed tests of advantageous flexibility.

This study addressed the issue of reduced sensitivity for pooled samples by modifying qRT-PCR of the assay. Thus, statistical calculation of reduced sensitivity for pooled samples described earlier [36, 42] does not apply to the malaria molecular assays used herein. GUI programmes designed here focused on simplicity and ease for laboratory staff to identify various pooling sizes for each pooling scheme based on given prevalence rates of studies and correspond test numbers to consider adequate pooling strategies for large malaria field study samples.

Additional file 4: Fig. S2A, and B provide visual demonstrations of anticipated test numbers when employing varied pooling sizes for the two-stage hierarchy and the square matrix pooling schemes, respectively. Additional file 4: Table S3 exemplifies results of the three-stage hierarchy pooling scheme for a cohort of 1000 samples with varied prevalence rates (i.e., 0.1%, 0.5%, 2%, 5% 7%, and 10%). Generally, the optimal pooling size depends on the prevalence of the studied cohort. However, it also showed that for studies with a prevalence rate > 10%, utilizing pooling strategies does not significantly reduce the number of required test counts.

Additional factors to consider when selecting a pooling strategy include: assay sensitivity, the number of replicates per specimen, labour hours required to prepare pooled samples upfront, and turnaround time [29]. For example, the three-stage hierarchy strategy generally yields lowest test counts but requires more aliquots or greater volumes of each specimen to contribute two rounds of pooling and takes three runs of testing to complete the first pooling, the second pooling and deconvolution, which results in a longer turnaround time to complete processing. One advantage of square matrix pooling is less dilution of potential positive sample, but due to the complexity of the matrix pooling, robotic liquid handling devices are preferred to aliquot samples to create row pools and column pools [29]. For the UW qRT-PCR assay, the two-stage hierarchy pooling strategy was chosen for easiest pooling of large quantities of DBS samples that overrides the disadvantage of slightly more test counts compared to the three-stage hierarchy and square matrix pooling schemes. This approach described in the pilot study yielded results that matched a previous individual sample study but with shorter turnaround times and fewer required tests.

GUIs can be used to model test counts if positive cases are evenly distributed in pooled samples, or are more clustered, as anticipated in samples from field studies. For example, Hsiang et al*.* [40] carried out a multiple-step pooling scheme for a cohort of 891 DBS samples collected from child participants residing in Kampala, Uganda and identified 50 positive DBS samples present in 28 of 99 pools (i.e., 9 DBS/pool). It is likely that bias ranging from 0% (i.e., even distribution of positive cases) to 50% (i.e., positive cases clustered in half of tested pools) naturally occurs in collected samples from epidemiological and surveillance studies. The GUIs developed in this study allow researchers to explore possible pooling strategies for different patterns of distribution.

The World Health Organization reported that in 2018 an estimated 228 million people were infected by *Plasmodium* parasites resulting in 405,000 deaths, with children under 5 years of age accounting for 67% of these deaths [36]. Implementation of mass testing, tracking, and treatment of such infections is necessary to reduce transmission and deaths [37]. Control strategies and surveillance systems may be enhanced by use of more sensitive molecular assays. Pooled DBS sampling utilizing this qRT-PCR 18S rRNA assay provides one tool to efficiently test larger quantities of samples in support of such malaria control and elimination efforts.

## Conclusions

The *Plasmodium* 18S rRNA qRT-PCR assay was validated for DBS samples of varying parasite densities and adapted to reliably detect low-density samples from DBS pools. Malaria-specific GUI programmes were created to identify optimal two-stage hierarchy, three-stage hierarchy, and square matrix pooling schemes and to estimate maximum test counts for a given cohort with specified prevalence and clustering characteristics. This qRT-PCR assay and these pooling strategies may reduce costs and increase efficiency when testing large quantities of DBS samples in malaria field studies.

## Supplementary Information

**Additional file 1:** **Text S1.** Calculation of total test counts in the GUI for the square matrix pooling scheme. Total test count was the sum of tests for screening pools and individual deconvolution samples. Screening tests for pools was calculated as 2 × N × (number of samples/ N2). If an average of positive sample in each pool was calculated ≤ 1, deconvolution was determined not necessary (shown in Additional file 4: Fig. S3A); if the average of positive samples in each pool was calculated > 1, the Microsoft Excel GUI program assumed that the positive samples were distributed evenly in the matrix, shown in Additional file 4: Fig. S3B. The program counted the total number of pools as QUOTIENT plus one. If the number of positive cases were greater than the total number of pools, the program used function MOD to determine the number of pools having additional one positive case, while the difference would be the pools having one less positive case. The number of positive cases in the former pools were determined using the ROUNDUP function for the predicted positive cases divided by the total number of pools; the number of positive cases in latter pools were determined using the ROUNDDOWN function. The number of tests to deconvolute each positive pool was all members in positive Row pools or Column pools.

**Additional file 2:** Text S2. Primers/probes concentrations used for the UW qRT-PCR assay and DNA sequences. *Plasmodium falciparum* qRT-PCR employed 0.4 µM of PfDDT1451F21 (5’-GCGAGTACACTATATTCTTAT-3’), 0.4 µM of PfDDT1562R21 (5’-ATTATTAGTAGAACAGGGAAA-3’) and 0.1 µM of Pf probe (5'-[6-FAM]-ATTTATTCAGTAATCAAATTAGGAT-3'); for Pan *Plasmodium* qRT-PCR, they were 0.2 µM of PanDDT1043F19 (5’-AAAGTTAAGGGAGTGAAGA-3’), 0.2 µM of PanDDT1197R22 (5’-AAGACTTTGATTTCTCATAAGG -3’) and 0.1 µM of Pan probe (5'-[CAL Fluor Orange 560]-ACCGTCGTAATCTTAACCATAAACTATGCCGACTAG-3'); for TBP RT-PCR, they were 0.1 µM of TBP forward primer (5’-GATAAGAGAGCCACGAACCAC-3’), 0.1 µM of TBP reverse primer (5’- CAAGAACTTAGCTGGAAAACCC-3’) and 0.1 µM of TBP probe (5'-[Quasar 670]- CACAGGAGCCAAGAGTGAAGAACAGT-3').

**Additional file 3: File S1.** Estimation tool for maximum test counts for the two-stage hierarchy pooling scheme. **File S2.** Estimation tool for maximum test counts for square matrix pooling scheme. **File S3.** Estimation tool for maximum test counts for the three-stage hierarchy pooling scheme.

**Additional file 4: Table S1. **Measurements and percentages of detection date for varied concentrations of *P. falciparum* Armored RNA® in liquid and DBS samples by the standard assay. **Table S2.** Sensitivity of pan-*Plasmodium* qRT-PCR of simplified and standard assays for DBS samples. **Table S3.** Nearly optimized pooling sizes to yield lowest total test counts for a cohort of 1000 samples using three-stage hierarchy pooling schemes. **Table S4.** Total test counts for even and clustered (50%) distributions. **Figure S1.** Four different levels of *P. falciparum* 18S rRNA in DBS stored at four different tempeatures for one month. DBS labelled with the High, Moderate, Low, and Very Low controls contained 50 µL per spot of nominal 3.5 × 10^6^, 1.2 × 10^4^, 1.4 × 10^2^, and 5 × 10^2^ parasites/mL blood, respectively. Storage temperatures are as shown in the legend. Three spots were excised for each sample and each spot was tested twice. Mean and ± SD were plotted for results. Pf, *P. falciparum*; Pan, pan-*Plasmodium*. **Figure S2.** Estimated total test counts using the two-stage hierarchy and the square matrix pooling schemes. Test counts for a cohort of 1000 samples were calculated for varied pooling sizes and each five prevalence rates (i.e., 0.1, 0.5, 2, 5 and 10%) using the two-stage hierarchy pooling schemes (**A**) and the square matrix pooling scheme (**B**). Both figures do not show the extended trend for 0.1% prevalence rate that total test counts continue lower for larger pool sizes. **Figure S3.** Square matrix pooling scheme. The only positive case is identified by positive results of the first row pool and the first column pool (**A**). Even distribution (**B**) or clustered placement (**C**) of two positive cases occur in the materix.

## Data Availability

The data generated for this study is available from corresponding author on reasonable request.

## Abbreviations

CHMI: Controlled human malaria infection
CI: Confidence interval
DBS: Dried blood spot
GUI: Graphic user interface
rRNA: Ribosomal RNA
qRT-PCR: Quantitative reverse transcription polymerase chain reaction
SD: Standard deviation

## References

[1] WHO (2019). World malaria report 2019.

[2] Jiram AI, Ooi CH, Rubio JM, Hisam S, Karnan G, Sukor NM (2019). Evidence of asymptomatic submicroscopic malaria in low transmission areas in Belaga district, Kapit division, Sarawak. Malaysia Malar J.

[3] Wongsrichanalai C, Barcus MJ, Muth S, Sutamihardja A, Wernsdorfer WH (2007). A review of malaria diagnostic tools: microscopy and rapid diagnostic test (RDT). Am J Trop Med Hyg.

[4] Trampuz A, Jereb M, Muzlovic I, Prabhu RM (2003). Clinical review: severe malaria. Crit Care.

[5] Tangpukdee N, Duangdee C, Wilairatana P, Krudsood S (2009). Malaria diagnosis: a brief review. Korean J Parasitol.

[6] Jimenez A, Rees-Channer RR, Perera R, Gamboa D, Chiodini PL, González IJ (2017). Analytical sensitivity of current best-in-class malaria rapid diagnostic tests. Malar J.

[7] Das S, Peck RB, Barney R, Jang IK, Kahn M, Zhu M (2018). Performance of an ultra-sensitive *Plasmodium falciparum* HRP2-based rapid diagnostic test with recombinant HRP2, culture parasites, and archived whole blood samples. Malar J.

[8] Seilie AM, Chang M, Hanron AE, Billman ZP, Stone BC, Zhou K (2019). Beyond blood smears: qualification of *Plasmodium* 18S rRNA as a biomarker for controlled human malaria infections. Am J Trop Med Hyg.

[9] Cunningham JA, Thomson RM, Murphy SC, de la Paz AM, Ding XC, Incardona S (2020). WHO malaria nucleic acid amplification test external quality assessment scheme: results of distribution programmes one to three. Malar J.

[10] Wu L, van den Hoogen LL, Slater H, Walker PG, Ghani AC, Drakeley CJ (2015). Comparison of diagnostics for the detection of asymptomatic *Plasmodium falciparum* infections to inform control and elimination strategies. Nature.

[11] Gaye A, Bousema T, Libasse G, Ndiath MO, Konate L, Jawara M (2015). Infectiousness of the human population to *Anopheles arabiensis* by direct skin feeding in an area hypoendemic for malaria in Senegal. Am J Trop Med Hyg.

[12] Ouedraogo AL, Goncalves BP, Gneme A, Wenger EA, Guelbeogo MW, Ouedraogo A (2016). Dynamics of the human infectious reservoir for malaria determined by mosquito feeding assays and ultrasensitive malaria diagnosis in Burkina Faso. J Infect Dis.

[13] Slater HC, Ross A, Felger I, Hofmann NE, Robinson L, Cook J (2019). The temporal dynamics and infectiousness of subpatent *Plasmodium falciparum* infections in relation to parasite density. Nat Commun.

[14] Bousema T, Okell L, Felger I, Drakeley C (2014). Asymptomatic malaria infections: detectability, transmissibility and public health relevance. Nat Rev Microbiol.

[15] Ngasala B, Mutemi DD, Mwaiswelo RO (2019). Diagnostic performance of malaria rapid diagnostic test and microscopy compared with PCR for detection of *Plasmodium falciparum* infections among primary schoolchildren in Kibiti District, Eastern Tanzania: an area with moderate malaria transmission. Am J Trop Med Hyg.

[16] Girma S, Cheaveau J, Mohon AN, Marasinghe D, Legese R, Balasingam N (2019). Prevalence and epidemiological characteristics of asymptomatic malaria based on ultrasensitive diagnostics: a cross-sectional study. Clin Infect Dis.

[17] Cassol S, Salas T, Gill MJ, Montpetit M, Rudnik J, Sy CT (1992). Stability of dried blood spot specimens for detection of human immunodeficiency virus DNA by polymerase chain reaction. J Clin Microbiol.

[18] Amoah LE, Donu D, Abuaku B, Ahorlu C, Arhinful D, Afari E (2019). Probing the composition of *Plasmodium* species contained in malaria infections in the Eastern region of Ghana. BMC Public Health.

[19] Zainabadi K, Adams M, Han ZY, Lwin HW, Han KT, Ouattara A (2017). A novel method for extracting nucleic acids from dried blood spots for ultrasensitive detection of low-density *Plasmodium falciparum* and *Plasmodium vivax* infections. Malar J.

[20] Mharakurwa S, Daniels R, Scott A, Wirth DF, Thuma P, Volkman SK (2014). Pre-amplification methods for tracking low-grade *Plasmodium falciparum* populations during scaled-up interventions in Southern Zambia. Malar J.

[21] Taylor SM, Mayor A, Mombo-Ngoma G, Kenguele HM, Ouédraogo S, Ndam NT (2014). A quality control program within a clinical trial consortium for PCR protocols to detect *Plasmodium* species. J Clin Microbiol.

[22] Tilahun A, Yimer M, Gelaye W, Tegegne B (2020). Prevalence of asymptomatic *Plasmodium* species infection and associated factors among pregnant women attending antenatal care at Fendeka town health facilities, Jawi District, North west Ethiopia: a cross-sectional study. PLoS ONE.

[23] Grabias B, Essuman E, Quakyi IA, Kumar S (2019). Sensitive real-time PCR detection of *Plasmodium falciparum* parasites in whole blood by erythrocyte membrane protein 1 gene amplification. Malar J.

[24] Zhou Z, Mitchell RM, Kariuki S, Odero C, Otieno P, Otieno K (2016). Assessment of submicroscopic infections and gametocyte carriage of *Plasmodium falciparum* during peak malaria transmission season in a community-based cross-sectional survey in western Kenya, 2012. Malar J.

[25] Murphy SC, Daza G, Chang M, Coombs R (2012). Laser cutting eliminates nucleic acid cross-contamination in dried-blood-spot processing. J Clin Microbiol.

[26] Siner A, Liew ST, Kadir KA, Mohamad DSA, Thomas FK, Zulkarnaen M (2017). Absence of *Plasmodium inui* and *Plasmodium cynomolgi*, but detection of *Plasmodium knowlesi* and *Plasmodium vivax* infections in asymptomatic humans in the Betong division of Sarawak. Malaysian Borneo Malar J.

[27] Kaura T, Kaur J, Sharma A, Dhiman A, Pangotra M, Upadhyay AK (2019). Prevalence of submicroscopic malaria in low transmission state of Punjab: a potential threat to malaria elimination. J Vector Borne Dis.

[28] van Zyl GU, Preiser W, Potschka S, Lundershausen AT, Haubrich R, Smith D (2011). Pooling strategies to reduce the cost of HIV-1 RNA load monitoring in a resource-limited setting. Clin Infect Dis.

[29] Westreich DJ, Hudgens MG, Fiscus SA, Pilcher CD (2008). Optimizing screening for acute human immunodeficiency virus infection with pooled nucleic acid amplification tests. J Clin Microbiol.

[30] Lennon SE, Miranda A, Henao J, Vallejo AF, Perez J, Alvarez A (2016). Malaria elimination challenges in Mesoamerica: evidence of submicroscopic malaria reservoirs in Guatemala. Malar J.

[31] FDA Reviews of Qualified Biomarker: *Plasmodium* 18S rRNA/rDNA. https://www.fda.gov/drugs/biomarker-qualification-program/fda-reviews-qualified-biomarker-plasmodium-18s-rrnardna. Accessed 14 Apr 2020.

[32] Tang N, Pahalawatta V, Frank A, Bagley Z, Viana R, Lampinen J (2017). HIV-1 viral load measurement in venous blood and fingerprick blood using Abbott RealTime HIV-1 DBS assay. J Clin Virol.

[33] Dorfman R (1943). The detection of defective numbers of large populations. Annals Math Stat.

[34] Kwiatkowski TJ, Zoghbi HY, Ledbetter SA, Ellison KA, Chinault AC (1990). Rapid identification of yeast artificial chromosome clones by matrix pooling and crude lysate PCR. Nucleic Acids Res.

[35] Phatarfod RM, Sudbury A (1994). The use of a square array scheme in blood testing. Stat Med.

[36] McMahan CS, Tebbs JM, Bilder CR (2012). Two-dimensional informative array testing. Biometrics.

[37] Fajardo E, Metcalf CA, Chaillet P, Aleixo L, Pannus P, Panunzi I (2014). Prospective evaluation of diagnostic accuracy of dried blood spots from finger prick samples for determination of HIV-1 load with the NucliSENS Easy-Q HIV-1 version 2.0 assay in Malawi. J Clin Microbiol.

[38] Lim MD (2018). Dried blood spots for global health diagnostics and surveillance: opportunities and challenges. Am J Trop Med Hyg.

[39] Ben-Ami R, Klochendler A, Seidel M, Sido T, Gurel-Gurevich O, Yassour M (2020). Large-scale implementation of pooled RNA extraction and RT-PCR for SARS-CoV-2 detection. Clin Microbiol Infect.

[40] Hsiang MS, Lin M, Dokomajilar C, Kemere J, Pilcher CD, Dorsey G (2010). PCR-based pooling of dried blood spots for detection of malaria parasites: optimization and application to a cohort of Ugandan children. J Clin Microbiol.

[41] Johannessen A (2010). Dried blood spots in HIV monitoring: applications in resource-limited settings. Bioanalysis.

[42] Hou P, Tebbs JM, Wang D, McMahan CS, Bilder CR (2018). Array testing for multiplex assays. Biostatistics.

