# Supplementary material for: Application of dried blood spot sample pooling strategies for Plasmodium 18S rRNA biomarker testing to facilitate identification of infected persons in large-scale epidemiological studies
Source: Malar J. 2021 Oct 7;20:391. doi: 10.1186/s12936-021-03907-8 (PMC8499573; doi:10.1186/s12936-021-03907-8)

Table S1. Measurements and percentages of detection date for varied concentrations of *P. falciparum* Armored RNA® in liquid and DBS samples by the standard assay

***Pf* Armored RNA® Dilution**		**Liquid samples (log_{10} copies/mL blood)**				**DBS samples (log_{10} copies/mL blood)**			
Parasites /mL blood	Log_{10} copies/ mL blood	*Pf* qRT-PCR		Pan qRT-PCR		*Pf* qRT-PCR		Pan qRT-PCR	
		Mean	SD	Mean	SD	Mean	SD	Mean	SD
$2.4x10^5$	9.2	9.1	0.2	9.1	0.3	8.6	0.2	8.8	0.3
$2.8x10^4$	8.3	7.9	0.2	8.1	0.1	7.6	0.3	7.8	0.4
$2,8x10^3$	7.3	7.0	0.1	7.2	0.1	6.7	0.2	6.7	0.3
$2.8x10^2$	6.3	6.1	0.1	6.1	0.1	5.8	0.3	5.5	0.8
$2.8x10^1$	5.3	5.2	0.1	5.0	0.4	4.9	0.4	4.7	0.4
$1.7x10^1$	5.1	4.9	0.3	4.5	0.3	3.5 (4/6*)	n/a	4.3 (5/6*)	n/a
7	4.7	4.6 (1/3*)	n/a	3.7 (2/3*)	n/a	4.1 (1/6*)	n/a	3.7 (2/6*)	n/a

Pf: P. falciparum; Pan: pan-*Plasmodium.*
n/a, not applicable (SD not calculated for replicates of <100% detection rate)
SD reflects variation in two batches of samples measured by multiple operators; n=3-6 samples per concentration level.
* Average calculated for quantifiable samples only (numbers in parentheses are detected results/number of replicates tested)

Table S2. Sensitivity of pan-*Plasmodium* qRT-PCR of simplified and standard assays for DBS samples

Pf Armored RNA® DBS	Dilution	Log_{10} copies/mL blood	Log_{10} copies / 50-µL spot	Standard assay (log_{10} copies /50-µL spot)		Simplified assay (no TBP) (log_{10} copies /50-µL spot)	
				Mean#	SD	Mean+	SD
Level C	Neat	7.3	6.0	5.3	0.0	5.4	0.1
	1:3	6.8	5.5	4.9	0.1	4.8	0.1
	1:10	6.3	5.0	4.4	0.1	4.3	0.1
Level D	Neat	6.3	5.0	3.8	1.0	4.4	0.0
	1:3	5.8	4.5	3.4	0.7	3.5	0.5
	1:10	5.3	4.0	3.2	0.1	3.4	0.1
Level E	Neat	5.3	4.0	3.1	0.2	3.2	0.2
	1:3	4.8	3.5	0.0 (0/2*)	n/a	2.9	0.1
	1:10	4.3	3.0	0.0 (0/4*)	n/a	2.17 (4/8*)	n/a
Level F	Neat	5.1	3.8	3.0 (2/3*)	n/a	3.2	0.2
	1:3	4.6	3.3	2.6 (1/3*)	n/a	2.6 (4/5*)	n/a

	1:10	4.1	2.8	0.0 (0/6*)	n/a	1.7 (5/10*)	n/a

Pf: P. falciparum; n/a, not applicable (SD not calculated for replicates of <100% detection rate)

[#] n=2-6 replicates per dilution

+ n=4-10 replicates per dilution

* Average calculated for quantifiable samples only (numbers in parentheses are detected results/number of replicates tested)

Table S3. Nearly optimized pooling sizes to yield lowest total test counts for a cohort of 1,000 samples using three-stage hierarchy pooling schemes.

Prevalence rate	Number of positive cases	Pool size for the first step	Pool size for the second step	Total test count
0.1%	1	25	5	50
0.5%	5	25	5	90
2%	20	10	4	230
5%	50	6	3	417
7%	70	5	2	515
10%	100	4	2	650

Table S4. Total test counts for even and clustered (50%) distributions

Prevalence rate	Number of positive cases	Three-stage hierarchy pooling schemes			Two-stage hierarchy pooling schemes		
		Total test counts for even distribution	Total test counts for clustered distribution*	% reduction	Total test counts for even distribution	Total test counts for clustered distribution^	% reduction
0.1%	1	50	41	18%	63	47	25%
0.5%	5	90	46	49%	141	106	25%
2%	20	230	130	43%	283	213	25%
5%	50	417	267	36%	450	350	22%
7%	70	515	410	20%	530	390	26%
10%	100	650	550	15%	633	483	24%

* All positive cases were clustered in half (50%) of the first pools.

^ All positive cases were clustered in half (50%) of the pools.

Figure S1. Four different levels of *P. falciparum* 18S rRNA in DBS stored at four different tempeatures for one month. DBS labelled with the High, Moderate, Low, and Very Low controls contained 50 µL per spot of nominal $3.5x10^6$, $1.2x10^4$, $1.4x10^2$, and $5x10^2$ parasites/mL blood, respectively. Storage temperatures are as shown in the legend. Three spots were excised for each sample and each spot was tested twice. Mean and +/-SD were plotted for results. Pf, *P. falciparum*; Pan, pan-*Plasmodium*.

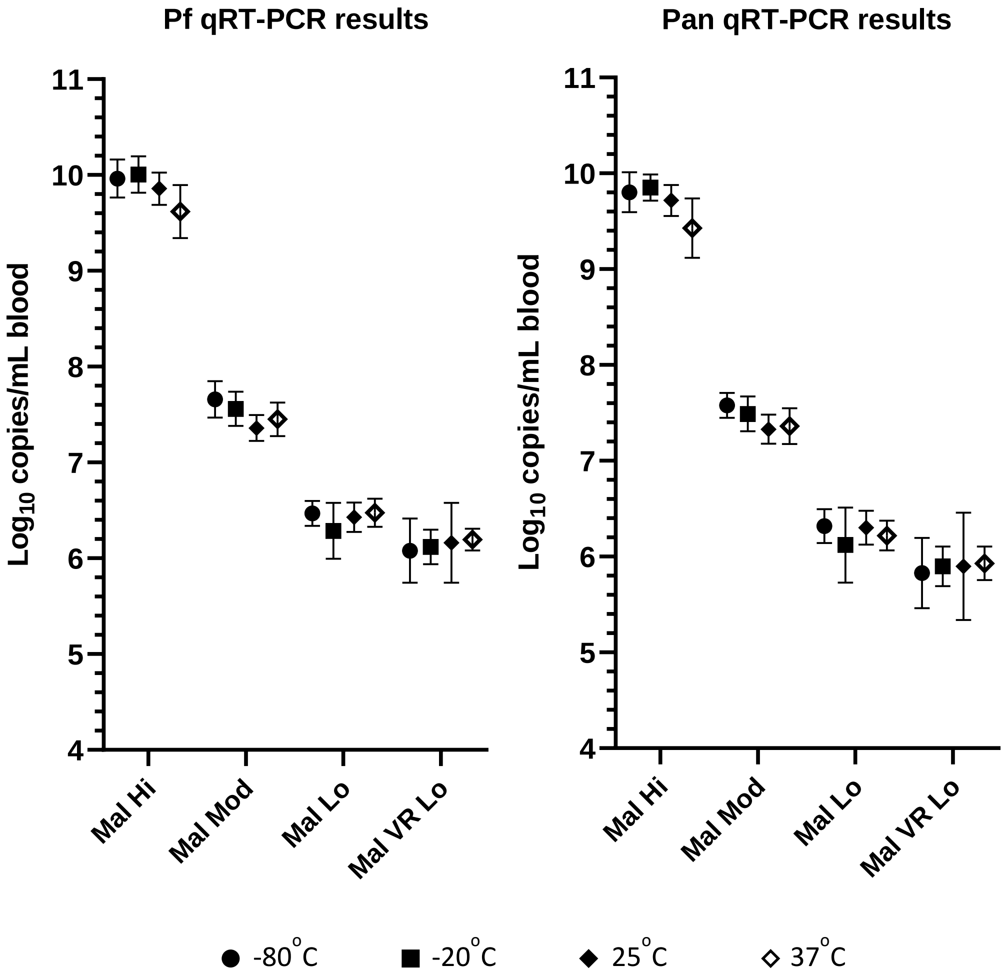

Figure S2. Estimated total test counts using the two-stage hierarchy and the square matrix pooling schemes. Test counts for a cohort of 1,000 samples were calculated for varied pooling sizes and each five prevalence rates (i.e., 0.1%, 0.5%, 2%, 5% and 10%) using the two-stage hierarchy pooling schemes (**A**) and the square matrix pooling scheme (B). Both figures do not show the extended trend for 0.1% prevalence rate that total test counts continue lower for larger pool sizes.

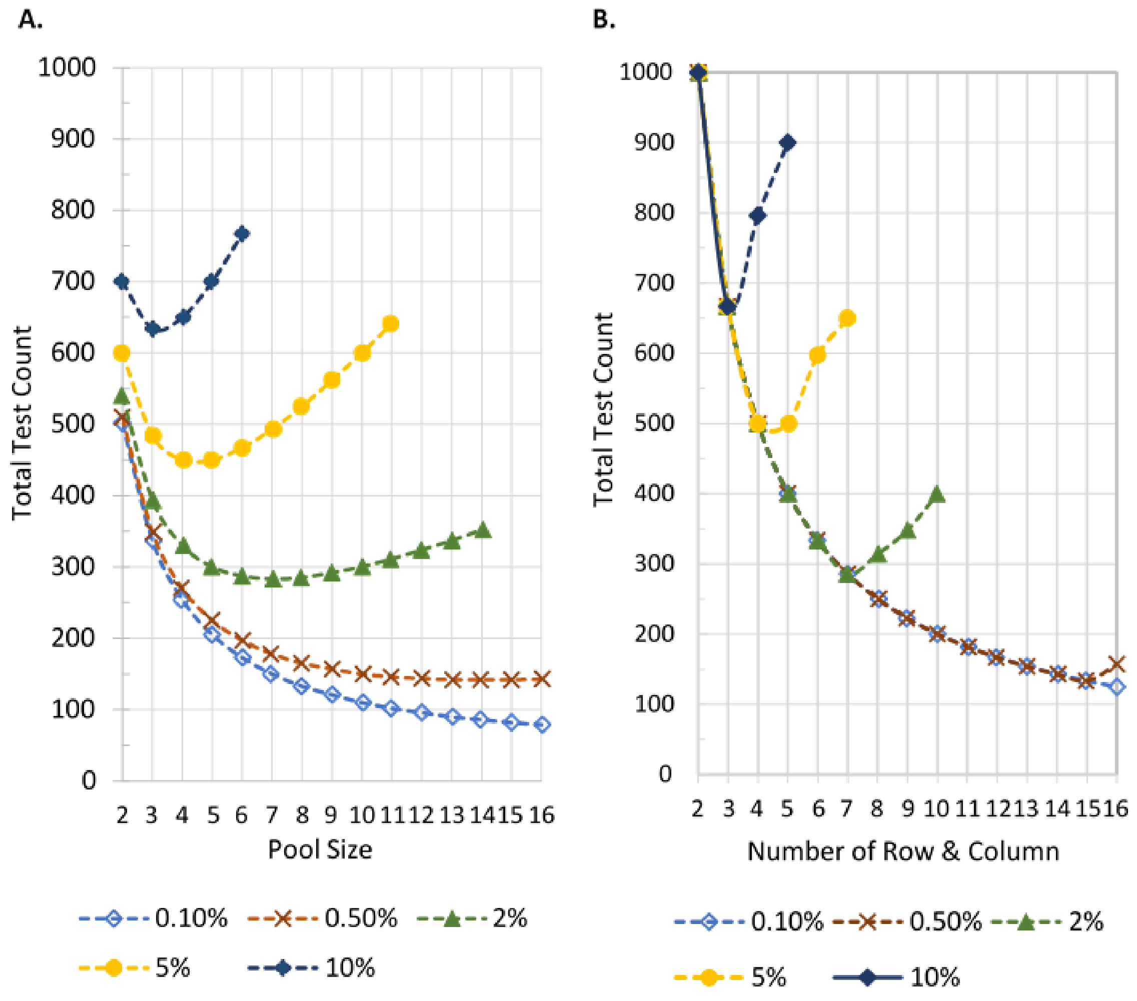

Figure S3. Square matrix pooling scheme. The only positive case is identified by positive results of the first row pool and the first column pool (A). Even distribution (B) or clustered placement (C) of two positive cases occur in the materix.

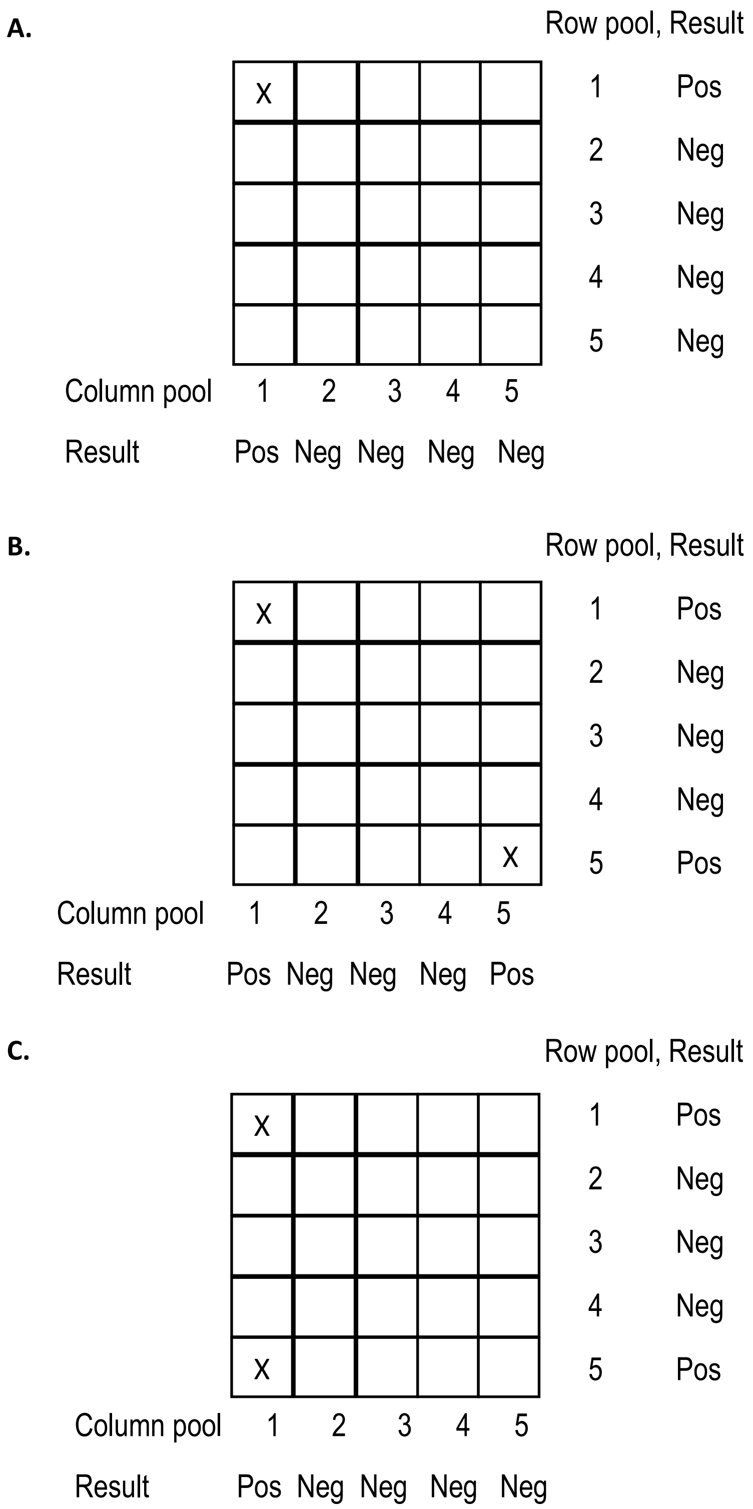

Supplement: Supplementary file 4 — Additional file 4: Table S1. Measurements and percentages of detection date for varied concentrations of P. falciparum Armored RNA® in liquid and DBS samples by the standard assay. Table S2. Sensitivity of pan-Plasmodium qRT-PCR of simplified and standard assays for DBS samples. Table S3. Nearly optimized pooling sizes to yield lowest total test counts for a cohort of 1000 samples using three-stage hierarchy pooling schemes. Table S4. Total test counts for even and clustered (50%) distributions. Figure S1. Four different levels of P. falciparum 18S rRNA in DBS stored at four different tempeatures for one month. DBS labelled with the High, Moderate, Low, and Very Low controls contained 50 µL per spot of nominal 3.5 × 10^6^, 1.2 × 10^4^, 1.4 × 10^2^, and 5 × 10^2^ parasites/mL blood, respectively. Storage temperatures are as shown in the legend. Three spots were excised for each sample and each spot was tested twice. Mean and ± SD were plotted for results. Pf, P. falciparum; Pan, pan-Plasmodium. Figure S2. Estimated total test counts using the two-stage hierarchy and the square matrix pooling schemes. Test counts for a cohort of 1000 samples were calculated for varied pooling sizes and each five prevalence rates (i.e., 0.1, 0.5, 2, 5 and 10%) using the two-stage hierarchy pooling schemes (A) and the square matrix pooling scheme (B). Both figures do not show the extended trend for 0.1% prevalence rate that total test counts continue lower for larger pool sizes. Figure S3. Square matrix pooling scheme. The only positive case is identified by positive results of the first row pool and the first column pool (A). Even distribution (B) or clustered placement (C) of two positive cases occur in the materix. [file 12936_2021_3907_MOESM4_ESM.pdf]
